# Self-organized twist-heterostructures via aligned van der Waals epitaxy and solid-state transformations

**DOI:** 10.1038/s41467-019-13488-5

**Published:** 2019-12-04

**Authors:** Peter Sutter, Rina Ibragimova, Hannu-Pekka Komsa, Bruce A. Parkinson, Eli Sutter

**Affiliations:** 1 Department of Electrical & Computer Engineering, University of Nebraska-Lincoln, Lincoln, NE 68588 United States; 2 Department of Applied Physics, Aalto University, P.O. Box 11100, FI-00076 Aalto, Finland; 3 Department of Chemistry and School of Energy Resources, University of Wyoming, Laramie, WY 82071 United States; 4 Department of Mechanical & Materials Engineering, University of Nebraska-Lincoln, Lincoln, NE 68588 United States

**Keywords:** Synthesis and processing, Two-dimensional materials, Microscopy, Nanoscale materials

## Abstract

Vertical van der Waals (vdW) heterostructures of 2D crystals with defined interlayer twist are of interest for band-structure engineering via twist moiré superlattice potentials. To date, twist-heterostructures have been realized by micromechanical stacking. Direct synthesis is hindered by the tendency toward equilibrium stacking without interlayer twist. Here, we demonstrate that growing a 2D crystal with fixed azimuthal alignment to the substrate followed by transformation of this intermediate enables a potentially scalable synthesis of twisted heterostructures. Microscopy during growth of ultrathin orthorhombic SnS on trigonal SnS_2_ shows that vdW epitaxy yields azimuthal order even for non-isotypic 2D crystals. Excess sulfur drives a spontaneous transformation of the few-layer SnS to SnS_2_, whose orientation – rotated 30° against the underlying SnS_2_ crystal – is defined by the SnS intermediate rather than the substrate. Preferential nucleation of additional SnS on such twisted domains repeats the process, promising the realization of complex twisted stacks by bottom-up synthesis.

## Abstract (web summary)

Vertically stacked twisted layers of two-dimensional materials can trigger exciting fundamental physics. Here, authors report controlled growth of 30° twisted few-layer SnS_2_ over SnS_2_ via van der Waals epitaxy of an SnS intermediate and its transformation in the presence of excess sulfur.

## Introduction

Van der Waals (vdW) heterostructures promise functional materials by integration of two-dimensional (2D) crystal building blocks^1^ while largely bypassing conventional structure- and lattice-matching requirements^2,3^. Recently, vertical heterostructures with controlled interlayer twist—a relative in-plane (azimuthal) rotation of one 2D crystal against the other—have attracted particular interest due to emerging electronic phenomena that are defined both by the atomic lattices and a periodic twist-moiré superlattice with substantially larger unit cell. For bilayer graphene with small magic-angle misorientation, this competition causes the emergence of electronic correlations and unconventional superconductivity^4,5^. In 2D semiconductor heterostructures, the interlayer coupling, electronic band structure, and optoelectronic properties depend on the twist angle^6–12^.

Mechanical stacking^13–15^ has been the preferred approach for fabricating vdW heterostructures^6,7^, as well as stacks with controlled static^16–18^ or dynamically adjustable interlayer twist^19^. But factors such as a lack of scalability as well as concerns about uniformity and possible interface contamination are motivating a search for bottom-up synthesis methods for twisted vdW heterostructures. There are two fundamentally interesting regimes for which synthesis approaches need to be developed: (i) Precise small-angle interlayer twists, which cause moiré patterns that give rise to a modulation of the electronic structure within large unit cells whose lateral dimensions can exceed 100 nm; and (ii) high-angle twists. In both cases, the realization of a controlled interlayer orientation during growth is hindered by the strong tendency of 2D crystals to stack in their equilibrium registry, i.e., azimuthally aligned without any twist, both for homo-stacks of the same or hetero-stacks of different but isostructural 2D materials^20–29^. The affinity toward equilibrium stacking makes it particularly challenging to realize small interlayer twist angles. In recent work, we showed that architectures that depart from the usual 2D vdW heterostructure geometry—specifically layered nanowires with Eshelby twist—can spontaneously yield self-organized twist moirés with small twist angles that are tunable via the nanowire diameter^30^.

For larger interlayer twists, the weak interaction between layered crystals can enable a different avenue toward the bottom-up synthesis of twist heterostructures via a two-step process in which the synthesis of an intermediate 2D (or 3D) crystalline phase (*B*) on a layered substrate (*A*) is followed by the conversion of the intermediate to a final phase *A*^rot^ with a defined azimuthal rotation relative to the substrate. Such a two-step process has recently been used for the creation of a dodecagonal quasicrystal in 30° twisted bilayer graphene^31^. In this case, monolayer h-BN served as an intermediate 2D crystal that grows on epitaxial graphene/SiC under 30° rotation, and a high-temperature annealing step replaced this template by a graphene layer with the same orientation, i.e., 30° twist relative to the underlying graphene. While this demonstration was based on a substitution between isostructural 2D honeycomb crystals, a similar strategy could be extended to other 2D/layered materials, such as metal chalcogenide semiconductors as well as intermediates that are not isotypic with the substrate. The viability of this generalized approach depends on two key requirements: Growth of the intermediate *B* has to involve a fixed azimuthal registry of *B* relative to the substrate *A*; and in the final solid-state transformation *B* → (twisted) *A*^rot^, the azimuthal orientation of the resulting crystal should be determined by the lattice structure of the intermediate rather than the substrate.

Here, we demonstrate the realization of this concept for layered tin chalcogenide semiconductors. For these materials, several stable layered crystal phases exist with different chalcogen content and prior work has demonstrated the conversion from chalcogen-rich trigonal SnS(e)_2_ dichalcogenide phases to orthorhombic SnS(e) monochalcogenides by generation of chalcogen vacancies, either thermally^32^, by electron irradiation^33^, or plasma exposure^34^. For the bottom-up growth of twisted vdW heterostructures, a bulk SnS_2_ single crystal plays the role of the layered substrate, *A*. The intermediate 2D crystal phase, *B*, is ultrathin SnS grown by vdW epitaxy. In situ low-energy electron microscopy (LEEM) during growth allows us to identify the sequence of azimuthally aligned vdW epitaxy and spontaneous solid-state transformations that ultimately gives rise to self-organized SnS_2_ twist heterostructures and incipient complex architectures, such as periodic vertical stacks with multiple twisted vdW interfaces.

## Results and discussion

**Fig. 1:**
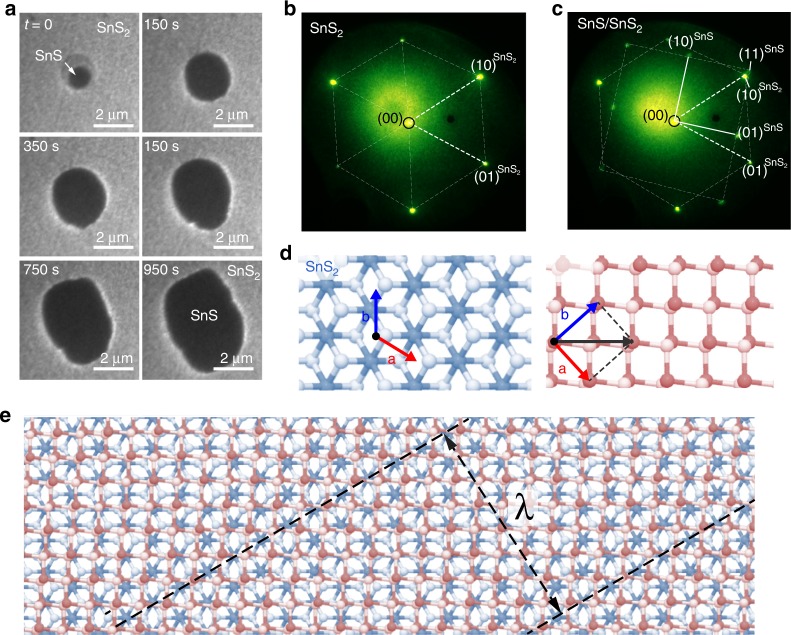
Direct growth of SnS/SnS_2_ heterostructures at *T* = 320 °C. **a** Real-time image sequence during deposition of SnS onto single-crystalline SnS_2_. Imaging electron energy: *E* = 4.3 eV. **b** Micro-LEED pattern of the SnS_2_ substrate (*E* = 50 eV). **c** Micro-LEED pattern of the SnS/SnS_2_ heterostructure (*E* = 50 eV). Note the azimuthal alignment between the $\left( {11} \right)^{{\mathrm{SnS}}}$ reflection of SnS and the $\left( {10} \right)^{{\mathrm{SnS}}_2}$ reflection of SnS_2_. The measured lattice mismatch along this direction is ~8%; both lattices adopt their bulk lattice constants during the vdW epitaxy. **d** Models of the SnS_2_ and SnS lattices with the observed azimuthal alignment. **e** Overlay of the two structures. *λ* denotes the wavelength of the stripe moiré pattern generated between the SnS and SnS_2_ lattices (see also Supplementary Fig. 1)^48^.

SnS was evaporated by congruent sublimation of intact formula units (i.e., SnS molecules) from a stoichiometric SnS powder precursor^23^ onto freshly cleaved SnS_2_ single crystals (and other substrates, see below) while imaging the growth process in real time by LEEM. At temperatures below 280 °C and between 320–340 °C, nucleation and growth produces heterostructures of single-crystalline few-layer SnS with lateral size up to several μm on atomically flat SnS_2_ vdW substrates (Fig. 1a). Note that this geometry is the inverse of previously reported SnS_2_/SnS vertical heterostructures^35^. Selected-area low-energy electron diffraction (micro-LEED) was used to analyze the crystal structure and lattice registry. The substrate shows the hexagonal surface mesh of single-crystalline SnS_2_ (Fig. 1b) with two distinct sets of alternating intense and weaker diffraction spots, associated with $\left( {10} \right)^{{\mathrm{SnS}}_2}$ and $\left( {01} \right)^{{\mathrm{SnS}}_2}$ reciprocal lattice vectors^36^. Composite micro-LEED patterns of SnS domains and of the surrounding SnS_2_ (Fig. 1c) show monocrystalline few-layer SnS. In contrast to other substrates (graphite, graphene)^23^ where SnS grows with random orientation, vdW epitaxy on SnS_2_ locks the SnS domains into a well-defined azimuthal alignment, found in all heterostructures reported here. In reciprocal space, the azimuth of the $\left( {11} \right)^{{\mathrm{SnS}}}$ reflection of SnS aligns with the $\left( {10} \right)^{{\mathrm{SnS}}_2}$ reflection of SnS_2_. Consistent with a relatively weak interlayer interaction, which can lock the SnS layer into a well-defined azimuthal orientation with the underlying SnS_2_ lattice but is not strong enough to force lattice matching, there is no detectable strain and the measured in-plane lattice constants of SnS and SnS_2_ coincide with values reported for the respective bulk single crystals^37,38^. Figure 1d, e illustrate the observed real-space azimuthal alignment in the SnS/SnS_2_ heterostructures, where the projected Sn-S bonds in SnS align with one of three bond directions of the SnS_2_ lattice. This preferred orientation generates three possible SnS domain orientations, separated by 120° (see Supplementary Fig. 2). The growth process shown in Fig. 1 can be rationalized by density-functional theory (DFT) based nudged elastic band calculations (Supplementary Fig. 3). The DFT results support a picture in which SnS congruently sublimed from precursor powder adsorbs on the SnS_2_ surface, diffuses, and nucleates or is incorporated into SnS domains. On-surface SnS dissociation is inhibited by large energy barriers (close to 1 eV), while lower activation energies (0.2–0.4 eV) enable the facile SnS surface diffusion by rotation between different S-sites.

**Fig. 2:**
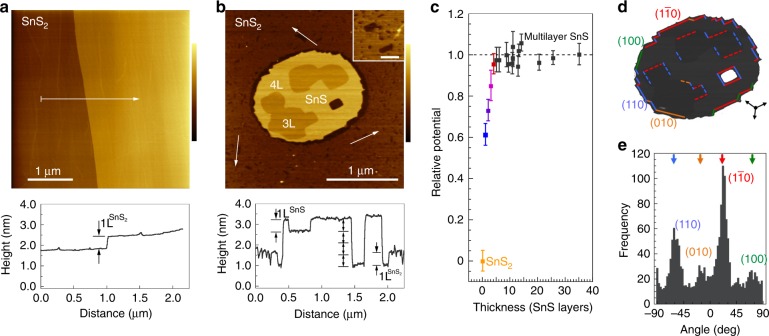
AFM of single-crystalline SnS_2_ and ultrathin SnS/SnS_2_. **a** AFM image of the SnS_2_ substrate with a monoatomic step. Bottom: Height profile along the line marked in the image. The step has height $1{\mathrm{L}}^{{\mathrm{SnS}}_2} \approx 0.6\,{\mathrm{nm}}$. Color scale: 3 nm. **b** AFM image of an ultrathin SnS/SnS_2_ vertical heterostructure. Note the abundant single-layer deep vacancy islands on the SnS_2_ surface, shown magnified in the inset (inset scale bar: 100 nm). Bottom: Height profile along the line marked in the image. Since the SnS/SnS_2_ vdW interface lies $1{\mathrm{L}}^{{\mathrm{SnS}}_2}$ below the average substrate surface, this particular SnS domain varies in height between $3 - 4{\mathrm{L}}^{{\mathrm{SnS}}}$. Color scale: 4 nm. **c** Kelvin probe force microscopy potential measurements of few-layer SnS relative to the SnS_2_ substrate, normalized to the potential of thicker (multilayer) SnS (see Supplementary Fig. 4). Error bars represent the full width at half maximum of Lorentzian fits to the distribution of surface potentials in areas of contstant SnS thickness. **d** Footprint of the SnS island shown in **c**. with color-coded edge facet segments. Arrows indicate the edge orientations of SnS_2_ vacancy islands marked in **b**. **e** Histogram of facet orientations determined from the AFM image shown in **b**. Source data are provided as a Source Data file.

Atomic force microscopy (AFM) has been used to further analyze the vertical tin chalcogenide heterostructures. Figure 2 shows AFM images of the SnS_2_ substrate prior to SnS growth, and of a SnS/SnS_2_ heterostructure. The freshly cleaved SnS_2_ substrate is flat, with atomic terraces separated by single-layer high steps (Fig. 2a). Following SnS deposition, AFM shows ultrathin few-layer SnS domains with lateral dimensions up to several μm, consistent with the LEEM results of Fig. 1 and evidence that synthesis on SnS_2_ avoids the strong tendency toward vertical growth of thicker SnS found for other vdW substrates^23^. The domain shown in Fig. 2b, for example, varies in thickness between 3–4 SnS layers ($1{\mathrm{L}}^{{\mathrm{SnS}}} \approx 0.56\,{\mathrm{nm}}$)^38^ and its vdW interface lies $1{\mathrm{L}}^{{\mathrm{SnS}}_2}$ below the average substrate surface. SnS flakes in this thickness range should allow the experimental realization of phenomena such as in-plane ferroelectric ordering^39–41^ and photostriction^42^ predicted for few-layer group IV monochalcogenides with odd number of layers. Surface potential measurements using Kelvin probe force microscopy (KPFM, see below) indeed show clear thickness-dependent properties (Fig. 2c, Supplementary Fig. 4). Such ultrathin SnS domains generally crystallize in a rounded shape bounded by micro-facets^43^. Also evident is the transformation of the atomically flat SnS_2_ surface into a patchwork of single-layer deep pits, where SnS_2_ was apparently removed during SnS growth (Fig. 2b, inset). Analysis shows that these single-layer deep vacancy islands cover about 20% of the SnS_2_ surface after SnS growth (see Supplementary Fig. 5). Comparison with the step orientation in the SnS_2_ pits, along with the azimuthal orientation determined by micro-LEED, identifies the SnS edges as majority $\left( {110} \right)$ and $\left( {1\bar 10} \right)$ facets, complemented by smaller segments of vicinal $\left( {100} \right)$ and $\left( {010} \right)$ facets (Fig. 2d, e). This domain shape is consistent with a recent analysis of kinetic growth shapes of thin SnS flakes^44^.

**Fig. 3:**
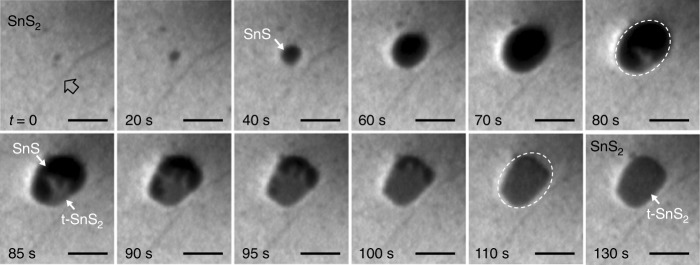
Phase conversion in SnS/SnS_2_ heterostructure growth at *T* = 300 °C. Real-time image sequence during deposition of SnS onto single-crystalline SnS_2_. The arrow at *t* = 0 marks a surface step on the SnS_2_ substrate. Transformation from SnS to t-SnS_2_ starts at *t* = 80 s. Note that the precise shape of the SnS flakes is difficult to image due to their large (~1 eV) difference in surface potential relative to the surrounding SnS_2_ surface and the resulting strong electric fields near the SnS edge. *E* = 5.0 eV. Scale bar: 1 μm.

**Fig. 4:**
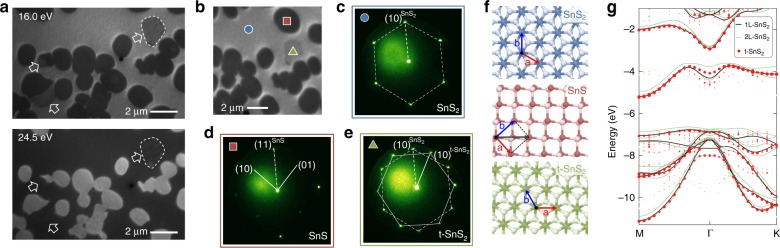
Identification of the phases obtained in SnS/SnS_2_ heterostructure growth at *T* = 300 °C. **a** LEEM image at 16.0 eV electron energy (top), and same sample area imaged at 24.5 eV electron energy (bottom). **b** LEEM image at 16.0 eV, showing the three different phases involved in growth at intermediate temperature (*T* ~ 300 °C). **c–e** Micro-LEED patterns obtained from the three distinct phases marked in **b**. **c**. SnS_2_ substrate; **d** Single-crystalline SnS; **e** Ultrathin twisted t-SnS_2_, rotated ~30° relative to the SnS_2_ substrate. **f** Top-views of the three structures, namely SnS_2_ (top), SnS aligned with the SnS_2_ substrate (center), and twisted t-SnS_2_, rotated ~30° relative to the SnS_2_ substrate (bottom). **g** Calculated band structures of single- and bilayer SnS_2_, in comparison with a 30° twisted t-SnS_2_ bilayer.

Whereas high and low substrate temperatures (*T*) favor direct growth of azimuthally aligned SnS/SnS_2_ heterostructures, intermediate *T* ~ 300 °C promotes an entirely different behavior, analyzed via real-time microscopy and diffraction (Figs. 3, 4). The initial nucleation and growth, identical to that shown in Fig. 1, again produces μm-sized SnS domains (Fig. 3; 0 ≤ *t* ≤ 70 s). Beginning at *t* = 80 s, the uniform contrast characteristic of SnS changes and a brighter phase (labeled t-SnS_2_) appears and progressively spreads across the entire domain (80–110 s), transforming the previously rounded SnS domain into a shape with extended straight facets (110–130 s). Repeated experiments with different growth conditions show no clear correlation of this transformation process with temperature (within the stated window, i.e., 280–320 °C), size of the SnS intermediate, or any features of the underlying substrate.

The product of the transformation is identified in Fig. 4. Imaging at different electron energy, *E*, shows the SnS_2_ substrate coexisting with two types of μm-scale domains with different *E*-dependent contrast (Fig. 4a). The analysis of these phases by micro-diffraction is shown in Fig. 4b–e. The diffraction patterns of hexagonal SnS_2_ (Fig. 4c) and orthorhombic SnS (Fig. 4d) are identical to Fig. 1, including the fixed azimuthal alignment of SnS on SnS_2_. Figure 4e shows diffraction from one of the domains transformed from SnS. The pattern is a superposition of two sets of hexagonal reflections, rotated relative to each other by 30°. From this result, we conclude that the converted domains consist of twisted t-SnS_2_, rotated in-plane by 30° relative to the substrate lattice; and the t-SnS_2_ is ultrathin, so that it contributes jointly with the underlying SnS_2_ to surface-sensitive diffraction. A further diffraction analysis provides evidence for superlattice reflections that arise from the twist moiré pattern of the hexagonal SnS_2_ crystals (Supplementary Fig. 6). Micro-LEED patterns on domains of 30° twisted t-SnS_2_/SnS_2_ show superlattice spots centered around the zone center and the first-order reflections of t-SnS_2_ (Supplementary Fig. 6a-d). Fast-Fourier transforms of such diffraction patterns reflect the emerging dodecahedral structural motifs associated with a Stampfli-tiling quasicrystal^45^ (Supplementary Fig. 6e, f) as realized recently in 30° twisted bilayer graphene^31^. The mechanism identified here, involving a solid-state transformation of a SnS intermediate, can also explain previously observed moiré patterns in SnS_2_ crystals synthesized by co-evaporation of Sn and S^46^. Consistent with previous work on twisted bilayer MoS_2_^7^, calculations show modifications of several key properties for 30° twisted SnS_2_. The twisted vdW gap of 6.22 Å is much larger than in equilibrium-stacked bilayer (5.87 Å) or bulk (5.82 Å) SnS_2_, and the interlayer binding energy decreases from 0.22 eV/unit cell in the aligned bilayer to 0.16 eV/unit cell in the twisted bilayer. Electronically, the twist decouples the layers at the vdW gap in t-SnS_2_ so that in a twisted bilayer they behave much like two monolayers, except for a few states around the Γ-point (Fig. 4g).

Evidently, a source of sulfur is required for the transformation of SnS to t-SnS_2_. The large SnS bond dissociation energy (467 kJ mol^−1^)^47^, congruent SnS sublimation^23^, and large on-surface dissociation energy (Supplementary Fig. 3; Supplementary Table 1) rule out SnS dissociation as the primary source of S, which instead comes from the slow thermal decomposition of the SnS_2_ substrate, explaining the formation of vacancy islands on the SnS_2_ surface (Fig. 2). An alternative source of excess sulfur is the presence of S-rich minority phases in the nominally pure SnS precursor powder, shown in recent work to spontaneously produce layered SnS-SnS_2_ core-shell heterostructures on mica vdW substrates^48^. Both the release of S from the SnS_2_ substrate and the supply of excess S from the precursor are consistent with the limited *T*-range in which the spontaneous SnS → t-SnS_2_ conversion is observed, as supported by DFT (Supplementary Fig. 3, Supplementary Fig. 7, Supplementary Fig. 8, Supplementary Table 2). At lower *T*, the thermal decomposition SnS_2_ → SnS + S and the incorporation of excess S into the growing SnS flakes are not activated, whereas S rapidly desorbs at higher *T*, likely via the formation of weakly bound S_x_ species (Supplementary Table 2). At intermediate *T*, here 280–320 °C, sulfur is available on the surface to spontaneously transform the growing SnS to twisted t-SnS_2_ (Supplementary Fig. 9). Note that the decomposition of the SnS_2_ surface layer via the thermally activated reaction SnS_2_ → SnS + S produces SnS and sulfur that are both mobile on the surface (Supplementary Fig. 3, Supplementary Fig. 7). Whereas the former adds to the deposited SnS, the adsorbed S contributes to the transformation of some of the SnS flakes into twisted t-SnS_2_. The limited amount of sulfur released from the substrate implies that only a small fraction of the SnS flakes can be converted to t-SnS_2_, as is indeed observed in Fig. 4. A higher yield of twisted SnS_2_ flakes may be obtained by supplying additional S from an external source. In this way, twisted dichalcogenide heterostructures can also realized on substrates that do not release substantial amounts of chalcogens (e.g., MoS_2_, WS_2_; see below).

**Fig. 5:**
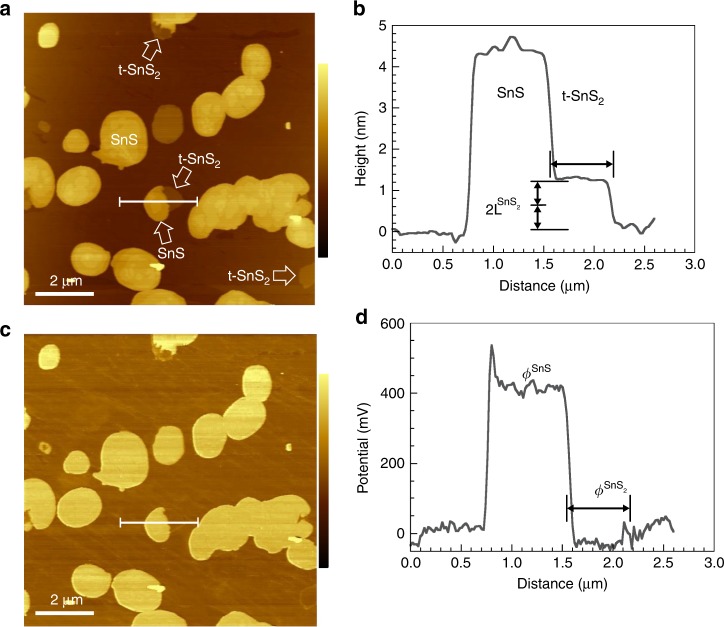
Ultrathin self-organized t-SnS/SnS_2_ twist heterostructures. **a** AFM image showing coexisting SnS/SnS_2_ and twisted t-SnS_2_/SnS_2_. Color scale: 15 nm. **b** Height profile along the line marked in **a**, identifying the t-SnS_2_ as a bilayer. **c** KPFM surface potential map of the area shown in **a**. Color scale: 1.0 V. **d** Potential profile along the same line trace as **b**, as marked in the maps in panels **a**. and **c**. In contrast to SnS, which shows a large potential difference, $\phi ^{SnS} - \phi ^{SnS_2} \cong + 400\,{\mathrm{mV}}$, relative to the surrounding SnS_2_, the surface potential of the 30° twisted t-SnS_2_ is indistinguishable from that of the SnS_2_ substrate. Surface potential measurements can thus unambiguously distinguish t-SnS_2_ from SnS.

AFM imaging confirms that the converted t-SnS_2_ is indeed ultrathin. Figure 5a shows coexisting SnS and t-SnS_2_ domains, where the latter are merely two atomic layers $\left( {2{\mathrm{L}}^{{\mathrm{SnS}}_2}} \right)$ thick (Fig. 5b). SnS and t-SnS_2_/SnS_2_ are clearly distinguished via their surface potential, measured by KPFM (Fig. 5c, d). Generally, the potential $\phi ^{{\mathrm{SnS}}}$of SnS is higher than that of the surrounding SnS_2_ substrate, with ${\mathrm{\Delta }}\phi = \phi ^{{\mathrm{SnS}}} - \phi ^{{\mathrm{SnS}}_2} \cong + 400\,{\mathrm{mV}}$ measured by KPFM in air. Twisted t-SnS_2_ domains show the same potential as the SnS_2_ substrate as expected due to their identical chemical nature and minimal potential shift due to twisted stacking. These findings are confirmed by local LEEM *I*–*V* measurements of the surface potential in ultrahigh vacuum (UHV, see Supplementary Fig. 10)^49^. Pristine samples again show a large positive potential of SnS relative to the surrounding SnS_2_ but a negligible difference between t-SnS_2_ and SnS_2_. For pristine SnS domains in UHV, ${\mathrm{\Delta }}\phi > + 1.0\,V$ (Supplementary Fig. 10, Supplementary Fig. 11). Air exposure changes both $\phi ^{{\mathrm{SnS}}}$ and $\phi ^{{\mathrm{SnS}}_2}$, reducing Δ*ϕ* to ~ 380 mV, consistent with the KPFM results. Annealing in UHV essentially recovers the pristine ${\mathrm{\Delta }}\phi \sim + 1.0\,V$, consistent with adsorption of ambient species, strongly bound on SnS but weaker on SnS_2_ (as shown by *T* for recovery of pristine potentials, SnS_2_: ~200 °C; SnS: ~300 °C).

**Fig. 6:**
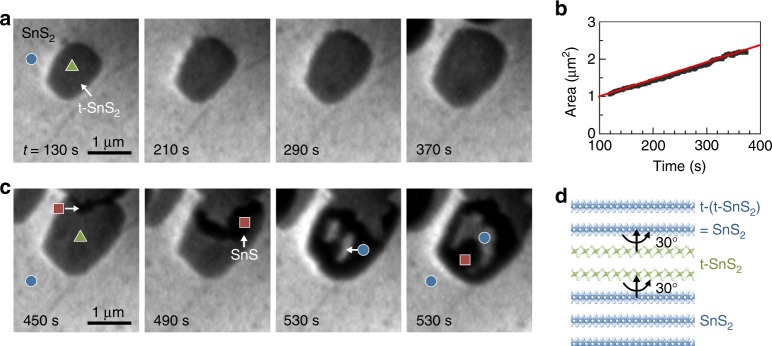
Cyclic twisted SnS_2_ growth and incipient SnS-mediated vertical twist-superlattices. **a** Real-time image sequence during continued deposition of SnS (following Fig. 3), showing the lateral expansion of the t-SnS_2_ domain. Imaging electron energy: *E* = 5.0 eV. **b** Analysis of the growth in projected area of the t-SnS_2_ domain. **c** SnS nucleation and spreading across the t-SnS_2_ footprint, followed by transformation to SnS_2_. **d** Schematic showing the twisted van der Waals stack resulting from cyclic SnS growth and transformation to SnS_2_.

We find a strong preference for SnS nucleation on ultrathin t-SnS_2_ domains during continued growth, which can give rise to a cyclic sequence of SnS growth and transformation to twisted SnS_2_. In situ microscopy illustrates this effect (Fig. 6). Starting with a 30°-rotated t-SnS_2_/SnS_2_ twist heterostructure, further SnS deposition causes the t-SnS_2_ domain to expand laterally, implying a continued reaction of SnS to SnS_2_ (Fig. 6a, b). This suggests that the t-SnS_2_ domains represent efficient sinks for adsorbed SnS, which attaches to the edges, captures S, and rapidly reacts to SnS_2_ at the microscopic level so that no SnS is detectable during real-time microscopy of the t-SnS_2_ domain expansion. Ultimately, SnS nucleates either homogeneously or, as shown here, heterogeneously near the coalescence point of two t-SnS_2_ domains (Fig. 6c; 450 s). The subsequent evolution involves the spreading of SnS confined to t-SnS_2_, followed by a renewed transformation to SnS_2_ (Fig. 6c; 490–530 s). Based on the characteristic contrast of SnS_2_, SnS, and t-SnS_2_, we find that SnS confined to t-SnS_2_ again undergoes a 30° azimuthal rotation relative to the underlying lattice as it transforms to SnS_2_. As illustrated in Fig. 6d, this additional twist brings this new SnS_2_ layer back into azimuthal alignment with the substrate. In this way, alternating ultrathin SnS_2_ with 0° and 30° twist is formed, suggesting that complex heterostructures, e.g., periodic vertical vdW superlattices with multiple twisted interfaces, may be realized by direct growth.

**Fig. 7:**
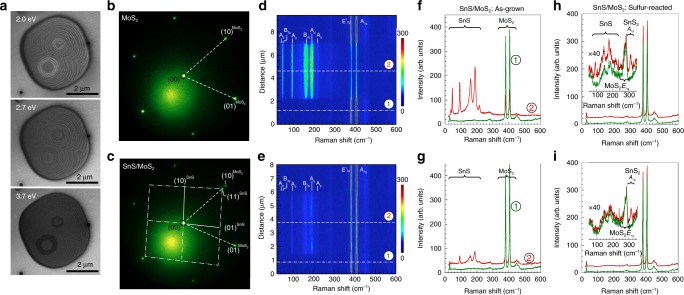
Extension to other materials systems – MoS_2_. **a** LEEM images of a few-layer SnS domain grown at *T* = 300 °C on a MoS_2_ substrate, imaged at different electron energies, *E*. **b** Micro-LEED pattern of the trigonal MoS_2_ substrate. **c** Micro-LEED pattern of SnS grown on MoS_2_. Note the two sets of diffraction spots, originating from SnS and MoS_2_, respectively, and the azimuthal alignment of the $\left( {11} \right)^{{\mathrm{SnS}}}$ reflection of SnS and the $\left( {10} \right)^{{\mathrm{MoS}}_2}$ reflection of MoS_2_. Both lattices adopt their bulk lattice constants during the vdW epitaxy. **d** Raman linescan of a thicker few-layer SnS flake with ~5 μm lateral size on MoS_2_, with the major Raman-active modes of both materials identified. **e** Raman linescan of an ultrathin SnS flake with ~5 μm lateral size on MoS_2_. **f** Raman spectra of the few-layer SnS flake (red) and of the nearby MoS_2_ substrate (green), extracted at locations shown in **d**. **g** Raman spectra of the ultrathin SnS flake (red) and of the nearby MoS_2_ substrate (green), extracted at locations shown in **e. h** Raman spectra of a few-layer SnS flake (red) and of the MoS_2_ substrate (green), following reaction with sulfur at 370 °C for 2 h. The inset shows the intensity of the main SnS Raman modes, along with an additional peak at 311 cm^−1^ due to the *A*_1g_ mode of SnS_2_. **i** Raman spectra of an ultrathin SnS flake (red) and of the MoS_2_ substrate (green), following reaction with sulfur at 370 °C for 2 h. The inset shows the absence of SnS Raman modes and a peak at 311 cm^−1^ due to the *A*_1g_ mode of SnS_2_, indicating complete conversion of the ultrathin SnS to SnS_2_.

To demonstrate the generality of the concepts identified here, i.e., a strong tendency toward azimuthally aligned vdW epitaxy in non-isotypic 2D chalcogenide semiconductors and the ability of transforming an aligned intermediate to realize twisted vdW stacks—we performed additional growth experiments involving SnS vdW epitaxy on MoS_2_ (Fig. 7, Supplementary Fig. 12) and WS_2_ (Supplementary Fig. 13) substrates. On MoS_2_, SnS growth at 300 °C produces ensembles of high-quality few-layer SnS flakes that expand to several μm lateral size (Supplementary Fig. 12) and exhibit a well-ordered layered morphology (Fig. 7a). Micro-LEED shows single-crystal diffraction patterns for both the MoS_2_ substrate (Fig. 7b) and the SnS flakes (Fig. 7c). Importantly, diffraction analysis shows the same azimuthal alignment for SnS on MoS_2_ as found for SnS on SnS_2_, namely the $\left( {11} \right)^{{\mathrm{SnS}}}$ reflection of SnS aligns with the $\left( {10} \right)^{{\mathrm{MoS}}_2}$ reflection of the MoS_2_ substrate. Likewise, growth on WS_2_ again locks the SnS into the same azimuthal registry with the substrate (Supplementary Fig. 13). While the in-plane orientation is fixed, the SnS flakes grow unstrained with their native in-plane lattice parameters on the different substrates.

In contrast to growth on SnS_2_, where some of the SnS flakes transform to t-SnS_2_, such a spontaneous transformation is not observed during growth on MoS_2_ or WS_2_, consistent with the absence of their thermal decomposition into stable Mo or W monochalcogenides. However, conversion of the deposited SnS to SnS_2_ can be realized by post-growth reaction with sulfur (see Methods), as shown by Raman spectroscopy analysis in Fig. 7d–i. Figure 7d, e shows Raman linescans of as-grown few-layer and ultrathin SnS flakes on MoS_2_. Uniform modes across the SnS flakes confirm their single-crystallinity^48^. Representative spectra show the characteristic *B*_3g_ and *A*_g_ modes of SnS along with intense *E*^*1*^_2g_ and *A*_1g_ peaks of the MoS_2_ substrate (Fig. 7f, g)^50^. In the as-grown flakes, the most intense vibrational mode of SnS_2_ (*A*_1g_, ~ 311 cm^−1^)^36^ is not detectable. Figure 7h, i shows Raman spectra from flakes on the same sample following a 2-hour exposure to sulfur vapor at 370 °C. After sulfurization, few-layer flakes show the appearance of the SnS_2_ *A*_1g_ peak with intensity similar to that of the SnS modes (Fig. 7h), indicating a partial transformation to SnS_2_ during annealing in S-vapor. For ultrathin sulfurized flakes, the SnS vibrational modes are no longer detectable but are replaced by the SnS_2_ *A*_1g_ mode, indicating a complete transformation of these SnS flakes to SnS_2_.

## Conclusions

From our combined findings, we can draw several conclusions. Firstly, azimuthally aligned vdW growth appears to be widespread, even for non-isotypic crystals such as orthorhombic SnS on trigonal SnS_2_, MoS_2_, and WS_2_ substrates. In cases where the support can release significant amounts of sulfur at the growth temperature, as is the case for SnS_2_ substrates, ultrathin aligned intermediate crystals can spontaneously transform to form twisted heterostructures. On substrates with limited sulfur release, a two-step process with post-growth sulfurization can achieve the same result. Our observations also suggest that exposure to additional sulfur may be used to increase the fraction of SnS flakes that transform to t-SnS_2_ during growth on SnS_2_ substrates. Finally, we found indications that the nucleation and transformation of an azimuthally aligned intermediate crystal phase may also govern the growth on non-chalcogenide substrates, if sulfur is provided in the vapor phase. As shown in Supplementary Figure 14, vapor transport growth of SnS on mica substrates with exposure to sulfur gives rise to two populations of oriented SnS_2_ flakes: A majority phase (~82% of all flakes) and a minority phase (~18%) rotated by 30°. This again supports a growth mechanism in which aligned SnS crystals ultimately transform into 30°-rotated t-SnS_2_. While the work reported here focused on transformations between 2D SnS and t-SnS_2_ crystals to realize the bottom-up synthesis of twisted vdW heterostructures, our results raise the possibility of using the sulfurization of 3D crystals, e.g., thin metal or metal oxide templates for forming twisted chalcogenide heterostructures. Future work needs to show if such 3D intermediates can be grown in a fixed azimuthal orientation with a vdW substrate, and if the azimuthal orientation of the final chalcogenide phase after transformation with sulfur will again be defined by the intermediate rather than the substrate.

## Methods

### Low-energy electron microscopy and micro-LEED of SnS growth on vdW substrates

High-quality SnS_2_ single crystals synthesized by the vertical Bridgman method were used as substrates. Additional layered supports included bulk MoS_2_ (extracted from natural minerals) and WS_2_ (synthetic, 2D Semiconductors). Prior to growth the layered substrates were mechanically cleaved and degassed at ~300 °C in ultrahigh vacuum (UHV). SnS was congruently evaporated from SnS powder (99.99%, Sigma–Aldrich) using a custom-built miniature Knudsen cell heated to 400-450 °C while observing the resulting surface processes in real time by bright-field LEEM. In situ LEEM, Micro-LEED, and other complementary measurements were performed in a modified Elmitec LEEM III microscope that allows observations at variable temperature in UHV (base pressure 2 × 10^−10^ Torr) and during sample exposure to gases or vapors (notably chalcogens or chalcogenides) with ~6 nm lateral and monolayer height resolution. Sample temperatures in LEEM were measured using a W-Re thermocouple spot-welded onto the sample support. Real-time image sequences were acquired at a rate of 1 frame per second and recorded at 1024 × 1024 pixels. Micro-LEED was performed in selected areas with ~1 μm lateral size. LEEM I–V data were acquired in real space with the full spatial resolution of the microscope; reported curves represent averages over areas with 200–400 nm lateral size.

### Post-growth sulfurization

SnS samples grown on MoS_2_ substrates were exposed to sulfur vapor in a separate quartz reactor implemented in a single-zone tube furnace with an additional external heating zone for sulfur. Sulfur powder (99.9995%, Alfa Aesar) was loaded into a quartz boat and as-grown SnS flakes on MoS_2_ were positioned in the center of the heating zone of the furnace. Following evacuation of the reactor a carrier gas mixture of Ar and H_2_ (ratio 98:2) was introduced at a flow rate of 50 sccm and a pressure of 76 Torr. The sulfur reservoir was heated to 80 °C (vapor pressure ~4 × 10^−4^ Torr) and sulfurization was carried out at a sample temperature of 370 °C for 2 h, followed by natural cooling to room temperature.

### Ex-situ measurements

AFM, phase mapping, and KPFM were carried out in tapping mode in air using a Veeco Multimode microscope with commercial SiN cantilevers or probes coated with thin metallic (Ru, ~10 nm) films. Raman spectroscopy was performed at room temperature in air in a Horiba Xplora Plus Raman microscope using a 100x objective at excitation wavelength of 532 nm and laser power of 0.168 μW. Computational methods are reported in the Supplementary Methods.

## Supplementary information

Supplementary Information

Description of Additional Supplementary Files

Supplementary Data 1

Supplementary Data 2

Supplementary Data 3

Supplementary Data 4

## Data Availability

All relevant data are available upon reasonable request from the corresponding author. The source data underlying Figs. 2c and 2e are provided as a Source Data file.
