# Supplementary material for: Self-organized twist-heterostructures via aligned van der Waals epitaxy and solid-state transformations
Source: Nat Commun. 2019 Dec 4;10:5528. doi: 10.1038/s41467-019-13488-5 (PMC6893034; doi:10.1038/s41467-019-13488-5)
Supplement: Supplementary file 1 — Supplementary Information [file 41467_2019_13488_MOESM1_ESM.pdf]

Supplementary Information

Self-Organized Twist-Heterostructures *via* Aligned van der Waals Epitaxy and Solid-State Transformations

Peter Sutter,[1,*] Rina Ibragimova,[2] Hannu-Pekka Komsa,[2] Bruce A. Parkinson,[3] and Eli Sutter[4]

[1]Department of Electrical & Computer Engineering, University of Nebraska-Lincoln, Lincoln, Nebraska 68588, United States
[2]Department of Applied Physics, Aalto University, P.O. Box 11100, FI-00076 Aalto, Finland
[3]Department of Chemistry and School of Energy Resources, University of Wyoming, Laramie, WY 82071, United States
[4]Department of Mechanical & Materials Engineering, University of Nebraska-Lincoln, Lincoln, Nebraska 68588, United States

1. Supplementary Figures

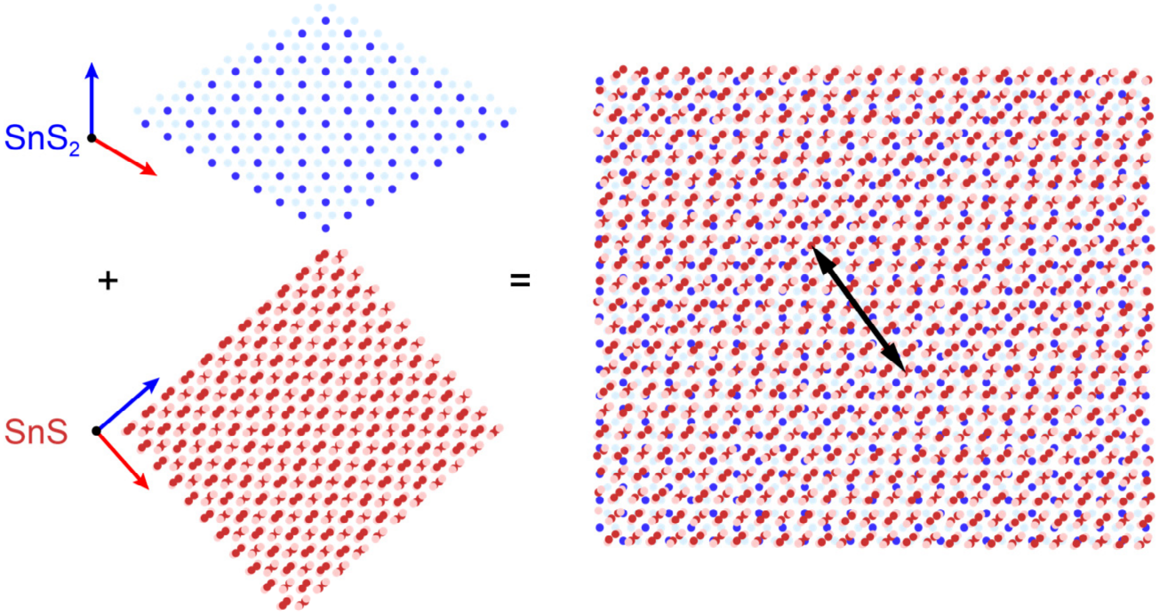

Supplementary Figure 1. Stripe moiré pattern formed between vertically stacked SnS and SnS_2 lattices. Left: Azimuthal orientation of SnS_2 and SnS as determined by micro-LEED on vertical SnS/SnS_2 heterostructures. Right: Resulting stripe moiré pattern. Arrow: Moiré period.

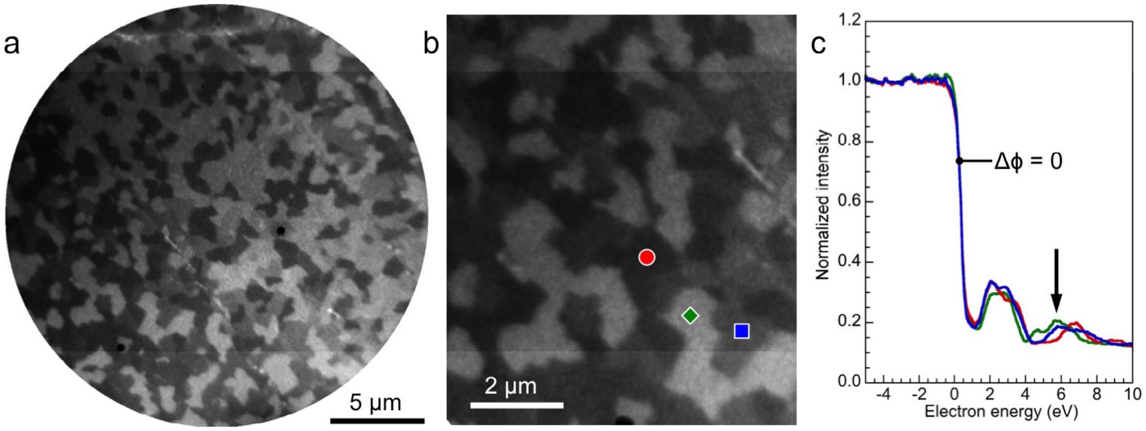

Supplementary Figure 2. Coexistence of three rotational SnS domains on hexagonal SnS_2. **a.** LEEM image of a closed (coalesced) few-layer SnS film on SnS_2. Imaging electron energy: E = 5.8 eV. **b.** Higher magnification view of a portion of the LEEM image in a. Colored symbols mark locations where *I-V* spectra were obtained. **c.** Local LEEM *I-V* characteristics, showing equal surface potentials ($\Delta\phi$ = 0) of the three rotational domains and small differences in the electron reflectivity at higher energies due to different orientation of the slightly tilted electron beam relative to the anisotropic SnS crystallites. For an explanation of LEEM *I-V* measurements of surface potentials, see Ref. 1.

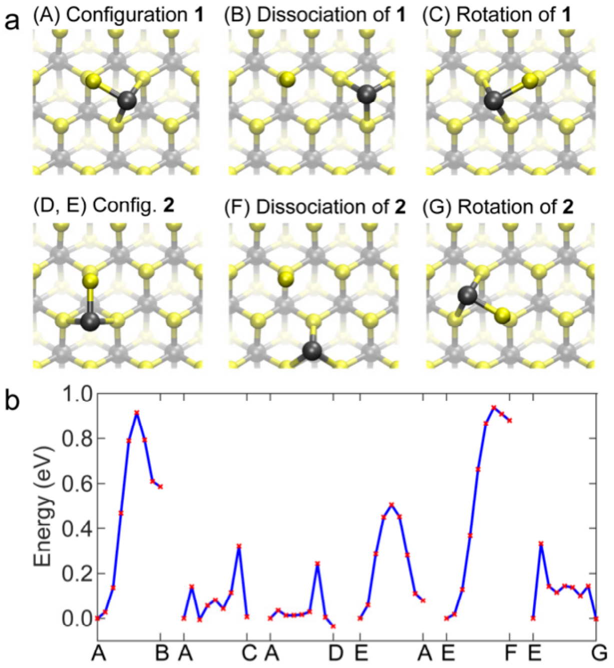

Supplementary Figure 3. Diffusion, rotation, and dissociation of SnS on SnS_2. a. Configurations of adsorbed SnS molecules on SnS_2. **b.** Nudged elastic band calculations of the energy barriers between the different states of adsorbed SnS shown in a. Note the large barriers preventing on-surface dissociation of adsorbed SnS.

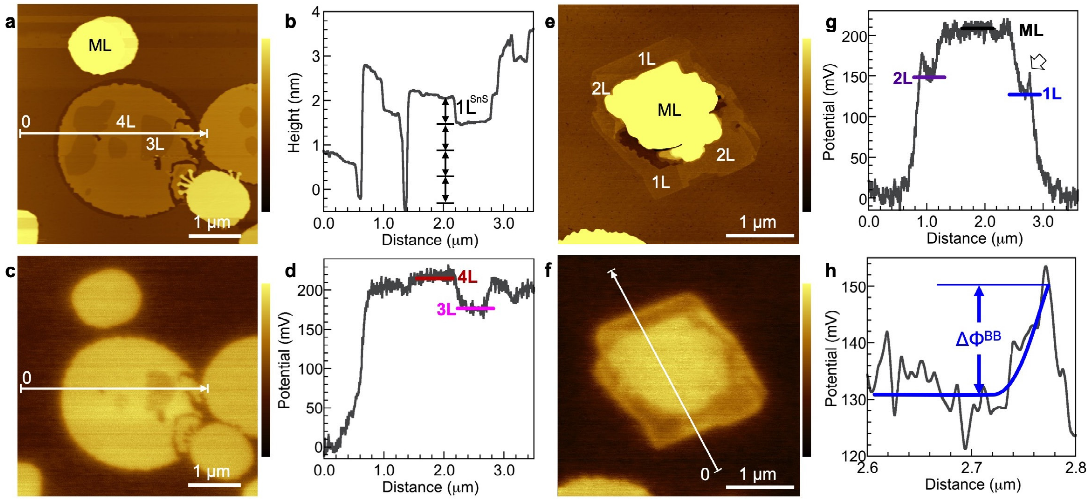

Supplementary Figure 4. AFM and KPFM analysis of ultrathin SnS domains on SnS_2. a. AFM image of a few-layer SnS/SnS_2 heterostructure with locally varying thickness (3-4 L, ML: multilayer). Color scale: 12 nm. **b.** Height profile along the line marked in a. **c.** KPFM potential map of the SnS domain shown in a. Color scale: 350 mV. **d.** Profile of the relative potential (difference $\phi_{\mathrm{SnS}} - \phi_{\mathrm{SnS_2}}$, normalized to $\phi_{\mathrm{ML\,SnS}}$ of multilayer SnS) along the line marked in c. **e.** AFM image of a multilayer SnS domain with ultrathin (1-2 L) fringe, grown on SnS_2. Color scale: 4 nm. **f.** Potential map of the SnS domain shown in e. Color scale: 250 mV. **g.** Profile of the relative potential along the line marked in f. **h.** Band-bending near the 2L SnS-SnS_2 interface (arrow in g).

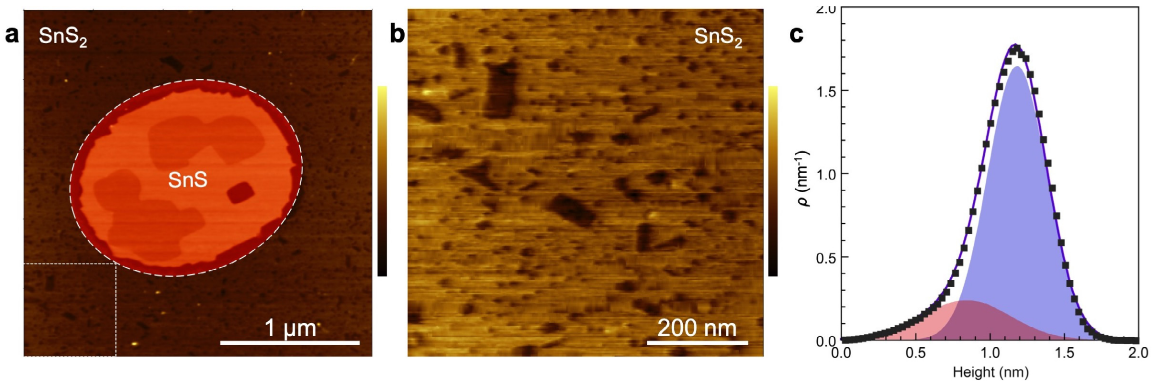

Supplementary Figure 5. Vacancy islands on the SnS_2 surface after SnS growth at 300°C. a. AFM image of few-layer SnS on SnS_2, as shown in Fig. 2 of the main text. Color scale: 4 nm. **b.** Magnified view of a section of the SnS_2 substrate next to the SnS island, marked by a dotted rectangle in a. Darker (low-lying) areas are single-layer deep vacancy islands embedded in the SnS_2 surface. Color scale: 1.8 nm. **c.** Height distribution analysis of the SnS_2 substrate outside the SnS island shown in a. (excluding the red shaded area). Peaks are Gaussian fits to the data: red – low-lying vacancy islands (20% of surface area); blue – majority of the SnS_2 surface (80% of surface area).

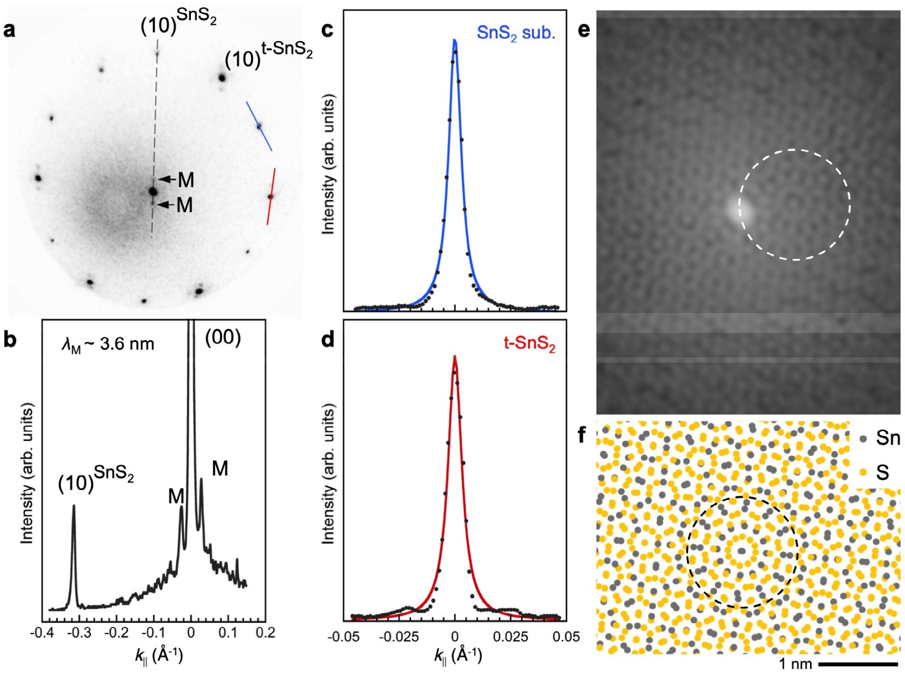

Supplementary Figure 6. Diffraction analysis of 30° twisted SnS_2 and twist moiré signatures. a. Micro-LEED pattern of a large domain of twisted t-SnS_2 grown on a SnS_2 single crystal. **b.** Intensity profile along the dashed line in a. Note the moiré superlattice spots, labeled M, near (00). The associated superlattice period λ_M is about 3.6 nm. **c.** Intensity profile of one of the diffraction spots of the SnS_2 substrate (blue line in a.). **d.** Intensity profile of one of the diffraction spots of the ultrathin twisted t-SnS_2 (red line in a.). **e.** Fourier transform of the diffraction LEED pattern of 30° twisted t-SnS_2 on SnS_2. **f.** Overlay of 0° and 30° rotated SnS_2 lattices, showing the same dodecagonal structural motifs found in e.

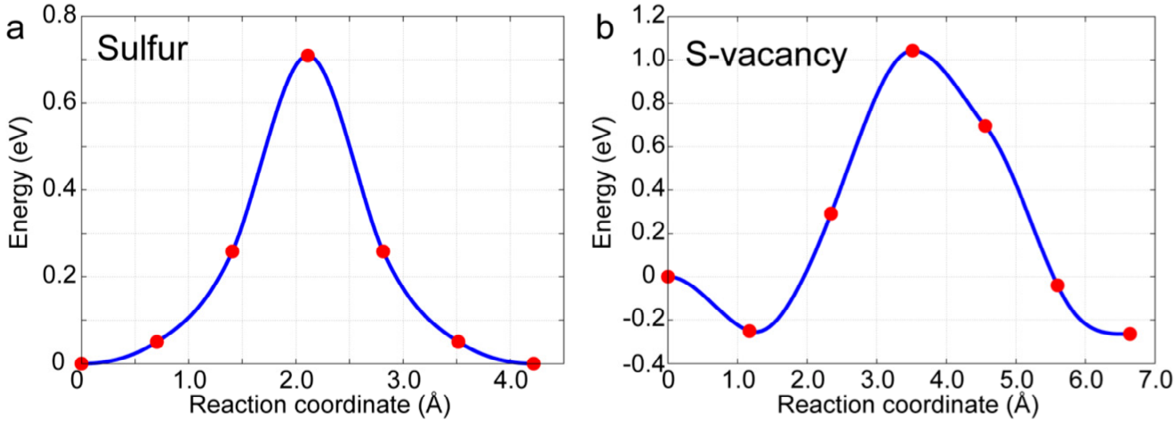

Supplementary Figure 7. Nudged elastic band calculations of S and S-vacancy migration barriers on SnS_2. a. Migration barrier of adsorbed S. **b.** S-vacancy migration barrier.

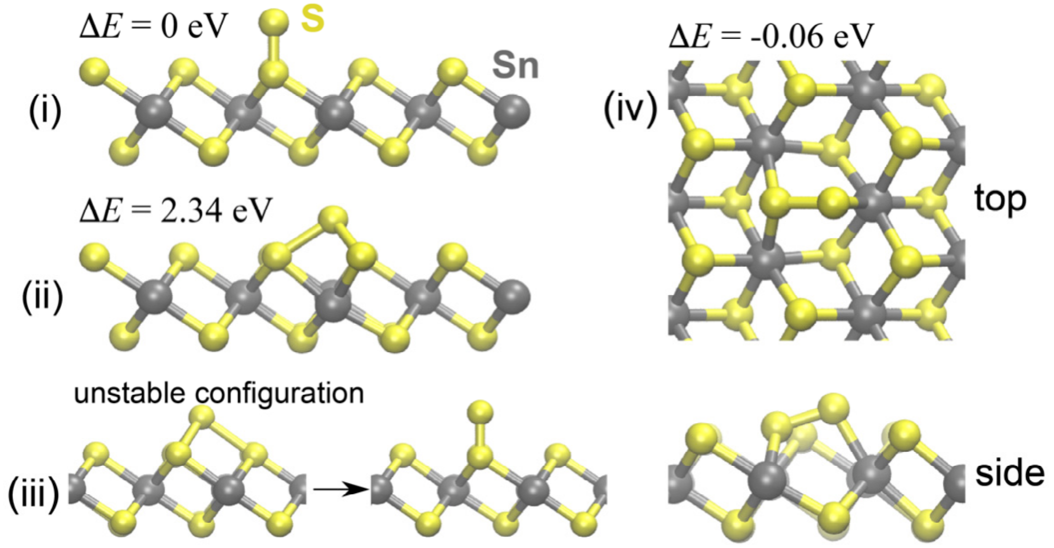

Supplementary Figure 8. S-adatom configurations on SnS_2. (i) S-top site. **(ii), (iii)** Hollow sites. **(iv)** 'Split interstitial' configuration (top view and side view). Energies ΔE are given for the different S-adatom configurations relative to top-site adsorbed S. Note the 'split interstitial' configuration, identified here as energetically favorable compared to the well-known top-site adsorption.

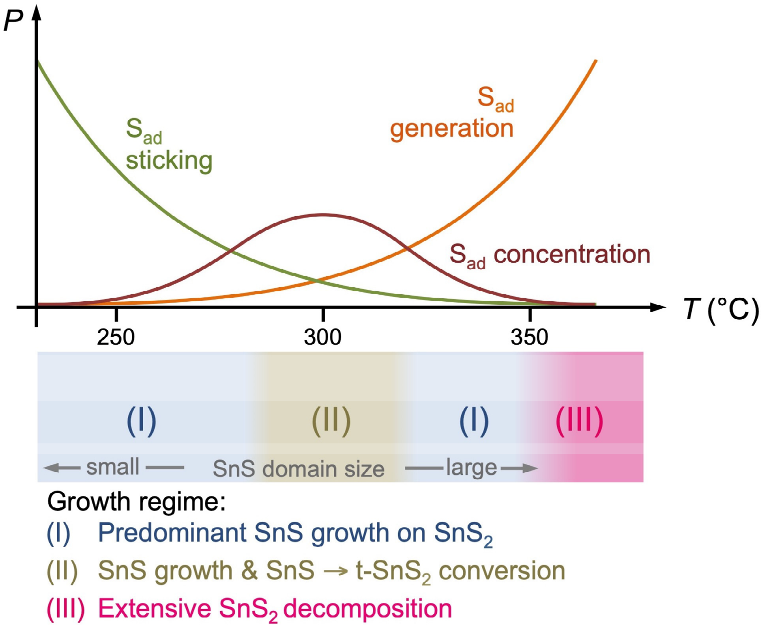

Supplementary Figure 9. Experimentally determined growth regimes for heterostructures formed by SnS deposition on crystalline SnS_2. Top. Schematic diagram illustrating the competition between the generation of surface-adsorbed sulfur (S_{ad}) by thermal decomposition of near-surface SnS_2 and thermal desorption reducing the sticking (residence time) of S_{ad}. Intermediate temperatures provide a maximum in the S_{ad} concentration. **Bottom.** Summary of the different growth regimes: (I) direct growth of SnS/SnS_2; (II) SnS growth and transformation to twisted t-SnS_2; (III) Extensive thermal decomposition of the SnS_2 surface.

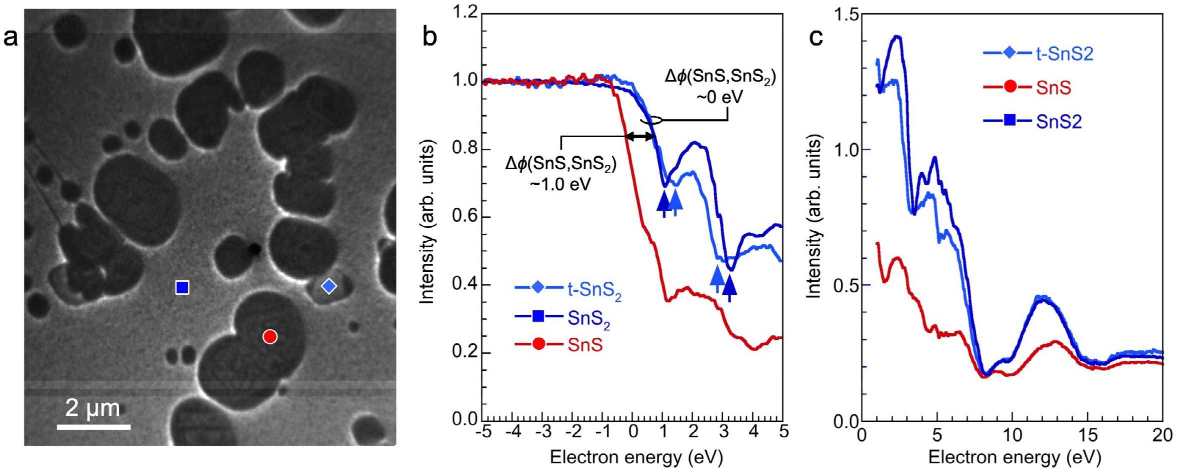

Supplementary Figure 10. UHV LEEM *I-V* of SnS, SnS_2, and twisted t-SnS_2 grown *in-situ*. a. LEEM image (electron energy: E = 5.0 eV) of a sample area containing both SnS (red circle) and t-SnS_2 (light blue diamond) on SnS_2 (blue square). **b., c.** LEEM *I-V* characteristics of the three phases, measured at the location of the corresponding symbols in (a). Note the substantial difference in surface potential ($\Delta\phi$) between SnS and SnS_2, but negligible potential difference between t-SnS_2 and SnS_2. The relative shift of the first *I-V* minima of SnS_2 and t-SnS_2 at ~1 eV and ~3 eV in (b) is consistent with the expected enlarged layer spacing at the twisted van der Waals interface.

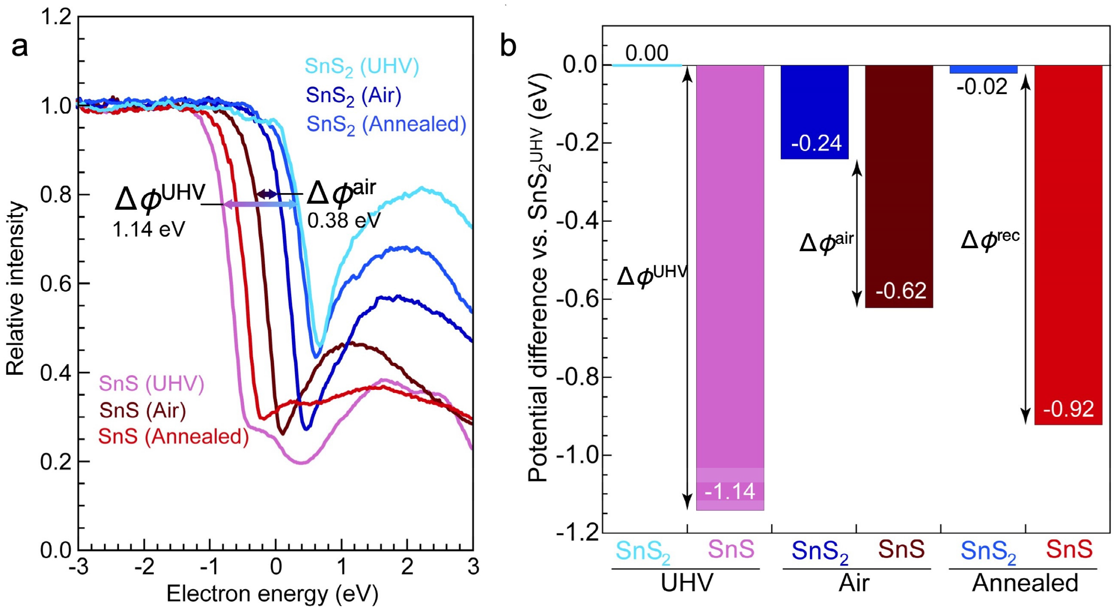

Supplementary Figure 11. Environment-induced variations in the surface potentials of SnS and SnS_2. a. LEEM surface potential measurements of SnS and SnS_2 after preparation in UHV, after exposure to air, and after subsequent annealing in UHV. **b.** Summary of the potential differences relative to pristine SnS_2. Note the decreased potential difference in the air-exposed sample ($\Delta\phi^{air}$), and the nearly complete recovery after annealing in UHV ($\Delta\phi^{rec} \approx \Delta\phi^{UHV}$).

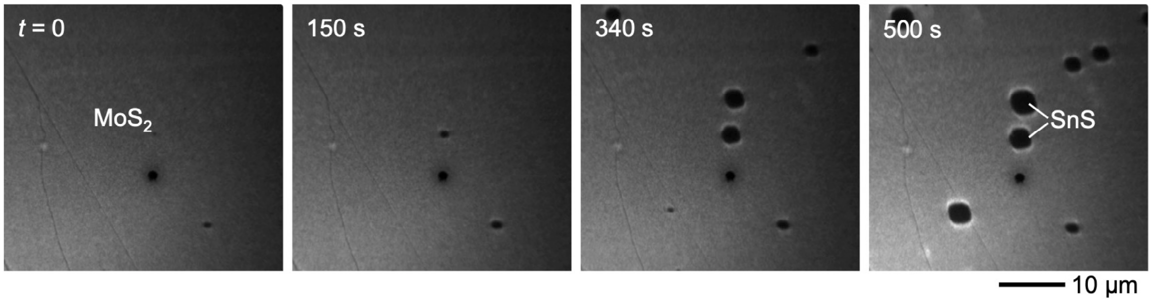

Supplementary Figure 12. Extension to other materials systems – MoS_2. Time-lapse series of LEEM images during the vdW growth of SnS on MoS_2 (elapsed time *t* in seconds). Faint lines are surface steps on the MoS_2 substrate. Growing dark regions are SnS flakes. Imaging electron energy: E = 3.0 eV.

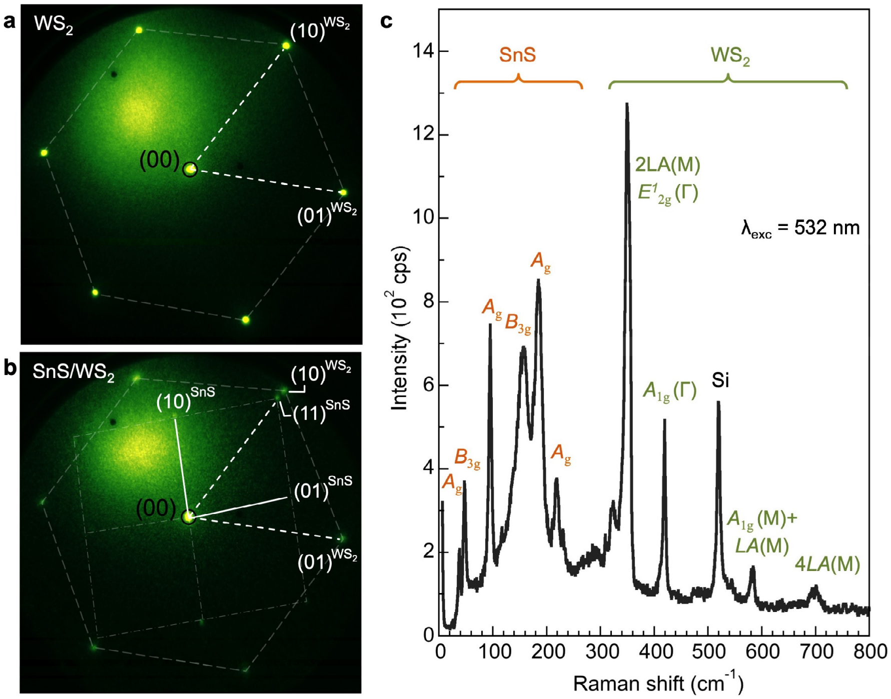

Supplementary Figure 13. Extension to other materials systems – WS_2. a. Micro-LEED pattern of the trigonal WS_2 substrate, showing the characteristic six-fold symmetry. **b.** Micro-LEED pattern of SnS grown on WS_2. Note the two sets of diffraction spots, originating from SnS and WS_2, respectively, and the azimuthal alignment of the $(11)^{\mathrm{SnS}}$ reflection of SnS and the $(10)^{\mathrm{WS_2}}$ reflection of WS_2. Both lattices adopt their bulk lattice constants during the vdW epitaxy. **c.** Raman spectrum of a SnS flake grown by vdW epitaxy on WS_2, showing the major Raman active modes of both SnS and WS_2.

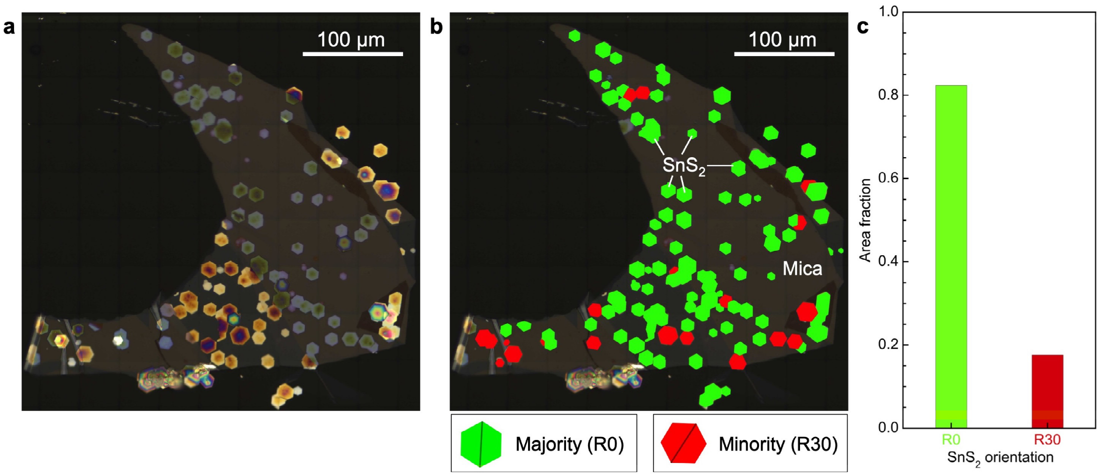

Supplementary Figure 14. Extension to other materials systems – mica. a. Optical microscopy of hexagonal SnS_2 flakes grown by SnS vapor transport in a sulfur atmosphere on a mica substrate **b.** Identification of two preferential orientations of SnS_2 flakes, a majority R0 oriented (green) and a minority 30° rotated (R30) phase (red). **c.** Analysis of the area fraction of R0 and R30 oriented SnS_2 flakes.

2. Supplementary Tables

Supplementary Table 1: Selected reaction energies, defined with their corresponding energy difference.

Reaction	Energy (eV)
$(SnS^{ad}) - (S^{adatom} + Sn^{adatom})$	-0.690
$(SnS^{ad} + S^{vacancy}) - (Sn^{adatom})$	2.386
$(SnS^{ad} + S^{vac}) - (Sn^{antisite} + S^{adatom})$	-0.594
$(S^{adatom} + S^{vacancy}) - ( )$	3.078
$(Sn^{adatom} + S^{vacancy}) - (Sn^{antisite})$	0.096

Supplementary Table 2: Adsorption energies of different species on the SnS_2 surface, calculated using $\Delta E_{\mathrm{ads}} = E_{\mathrm{SnS_2+ads}} + E_{\mathrm{SnS_2}} + E_{\mathrm{ads}}$, where $E_{\mathrm{SnS_2+ads}}$ is the total energy of the system with adsorbed unit, $E_{\mathrm{SnS_2}}$ is the total energy of the pristine SnS_2 slab, and E_{ads} denotes the energy of the adsorbate in vacuum.

	$\Delta E_{ads}^{pristine}$ (eV)	$\Delta E_{ads}^{V_S^0}$ (eV)
**S**	-2.521	-5.587
**S_2**	0.4102	
**SnS**	-0.593	-7.367

3. Supplementary Methods: Computational Details

Calculations were performed using the Vienna ab-initio simulation package (VASP)[2] together with projector augmented plane wave method (PAW).[3] The Perdew-Burke-Ernzerhof (PBE)[4] exchange-correlation functional has been used for all calculations, with the Van der Waals interactions included by a DFT-D3 correction proposed by Grimme.[5] The optimal plane-wave cutoff energy was chosen as 400 eV according to the convergence tests. The *k*-points set of 3x3x1 was chosen as optimal for slab calculations with 5x5x1 size of the supercell. A vacuum size of 22 Å in a supercell was chosen to prevent interactions between the slab and its periodical repetitions. The calculated in-plane lattice constant of SnS_2 is 3.67 Å. To determine the energy barriers of saddle points along the diffusion paths for adsorbed molecules, we used the climbing-image nudged elastic band (NEB) method.[6] The twisted heterostructure with minimal strain was built using supercell lattice vectors 7a and 8a+4b for the bottom and top layers, and then aligning them, leading to 49 and 48 units of SnS2 in the two layers, respectively.[7] This choice yields twist angle of exactly 30 degree and a strain of only 1 % for the top layer, while the lattice constant of the bottom layer is fixed to that of pristine SnS_2. Finally, the electronic structure is visualized using a band unfolding scheme.[7,8]

4. Supplementary References

1. Sutter, P. & Sutter, E. Microscopy of Graphene Growth, Processing, and Properties. *Advanced Functional Materials* **23**, 2617-2634 (2013).
2. Kresse, G. & Furthmüller, J. Efficient iterative schemes for ab initio total-energy calculations using a plane-wave basis set. *Physical Review B* **54**, 11169-11186 (1996).
3. Blöchl, P. E. Projector augmented-wave method. *Physical Review B* **50**, 17953-17979 (1994).
4. Perdew, J. P., Burke, K. & Ernzerhof, M. Generalized Gradient Approximation Made Simple. *Physical Review Letters* **77**, 3865-3868 (1996).
5. Grimme, S., Ehrlich, S. & Goerigk, L. Effect of the damping function in dispersion corrected density functional theory. *Journal of Computational Chemistry* **32**, 1456-1465 (2011).
6. Henkelman, G. & Jónsson, H. Improved tangent estimate in the nudged elastic band method for finding minimum energy paths and saddle points. *The Journal of Chemical Physics* **113**, 9978-9985 (2000).
7. Komsa, H.-P. & Krasheninnikov, A. V. Electronic structures and optical properties of realistic transition metal dichalcogenide heterostructures from first principles. *Physical Review B* **88**, 085318 (2013).
8. Popescu, V. & Zunger, A. Effective Band Structure of Random Alloys. *Physical Review Letters* **104**, 236403 (2010).
