# Supplementary material for: Self-organized twist-heterostructures via aligned van der Waals epitaxy and solid-state transformations
Source: Nat Commun. 2019 Dec 4;10:5528. doi: 10.1038/s41467-019-13488-5 (PMC6893034; doi:10.1038/s41467-019-13488-5)
Supplement: Supplementary file 2 — Description of Additional Supplementary Files [file 41467_2019_13488_MOESM2_ESM.pdf]

Description of Additional Supplementary Files

Supplementary Dataset 1 (file name: “Explanation_Fig2e.rtf”): Description of the approach used to analyse the edge angle distribution from an atomic force microscopy image of an ultrathin SnS flake on SnS_2 (Figure 2b of the paper), as shown in Figure 2d and Figure 2e.

Supplementary Dataset 2 (file name: “SummaryData_Fig2c.rtf”): Summary of the data points represented in Figure 2c of the paper, including flake thickness (in nanometers and rounded to SnS monolayers), relative potential (expressed as a fraction of the potential difference between the SnS_2 substrate and thick (multilayer) SnS/SnS_2), and full width at half maximum (FWHM) of the relative potential. Explanation of how these data points and the FWHM were obtained.

Supplementary Dataset 3 (file name: “20180712_sns_sns2_.013.tif”): Illustration of the thresholding procedure used to establish the edge angle histogram shown in Figure 2e of the paper. The compound image represents the original AFM image and overlaid (in red color) the same image after thresholding. The thresholded image was then analysed to obtain the edge angle distribution histogram of Figure 2e.

Supplementary Dataset 4 (file name: “Histogram_Fig2e.txt”): Raw data (abundance of edge orientation angles, in steps of 0.05 radians) plotted as a histogram in Figure 2e of the paper, obtained from an orientation analysis of the image shown in Supplementary Dataset 3 following the procedure described in Supplementary Dataset 1.
