# Supplementary material for: Self-organized twist-heterostructures via aligned van der Waals epitaxy and solid-state transformations
Source: Nat Commun. 2019 Dec 4;10:5528. doi: 10.1038/s41467-019-13488-5 (PMC6893034; doi:10.1038/s41467-019-13488-5)
Supplement: Supplementary file 3 — Supplementary Data 1 [file 41467_2019_13488_MOESM3_ESM.rtf]

The analysis of the SnS facet directions shown in Fig. 2e is based on the enclosed color coded AFM image (filename: “20180712_sns_sns2_.013.tif”). Edge orientations of the red shaded SnS flake were analyzed using the plugin OrientationJ in the software package ImageJ. The attached file “Histogram_Fig2e.txt” contains the results of this analysis, which are graphically represented in Fig. 2e of the paper.
