# Supplementary material for: Self-organized twist-heterostructures via aligned van der Waals epitaxy and solid-state transformations
Source: Nat Commun. 2019 Dec 4;10:5528. doi: 10.1038/s41467-019-13488-5 (PMC6893034; doi:10.1038/s41467-019-13488-5)
Supplement: Supplementary file 4 — Supplementary Data 2 [file 41467_2019_13488_MOESM4_ESM.rtf]

Fig. 2cMaterial	Height (nm)		Height	Relative Potential		Potential FWHMSnS2		0*			0 L*		0.00*				0.10SnS		0.6			1 L		0.61				0.10SnS		1.3			2 L		0.73				0.11SnS		2.0			3 L		0.85				0.15SnS		2.4			4 L		0.96				0.10SnS		3.1			5 L		0.98				0.11SnS		3.7			6 L		0.98				0.11SnS		5.6			9 L		1.00				0.12SnS		6.1			9 L		0.96				0.08SnS		7.0			11 L		0.96				0.10SnS		7.1			11 L		1.04				0.14SnS		7.1			11 L		0.99				0.88SnS		8.3			13 L		0.94				0.10SnS		8.5			13 L		1.02				0.06SnS		9.1			14 L		1.06				0.08SnS		13.3			20 L		0.96				0.08SnS		16.6			26 L		0.99				0.07SnS		21.0			35 L		1.00				0.10*Values for SnS2 are set to zero as reference.Potentials are obtained from KPFM data (e.g., Fig. 5, Fig. S4), either using line profiles or histograms  of surface potential within areas of constant thickness. Relative potentials are normalized to the mean surface potential of all analyzed SnS flakes with thickness greater than 5 L. FWHM values are obtained from Lorentzian fits to the measured potential distributions (obtained in areas of constant flake thickness) or standard deviation (for potentials measured from KPFM line profiles).
