# Supplementary figures and images for: Self-organized twist-heterostructures via aligned van der Waals epitaxy and solid-state transformations
Source: Nat Commun. 2019 Dec 4;10:5528. doi: 10.1038/s41467-019-13488-5 (PMC6893034; doi:10.1038/s41467-019-13488-5)

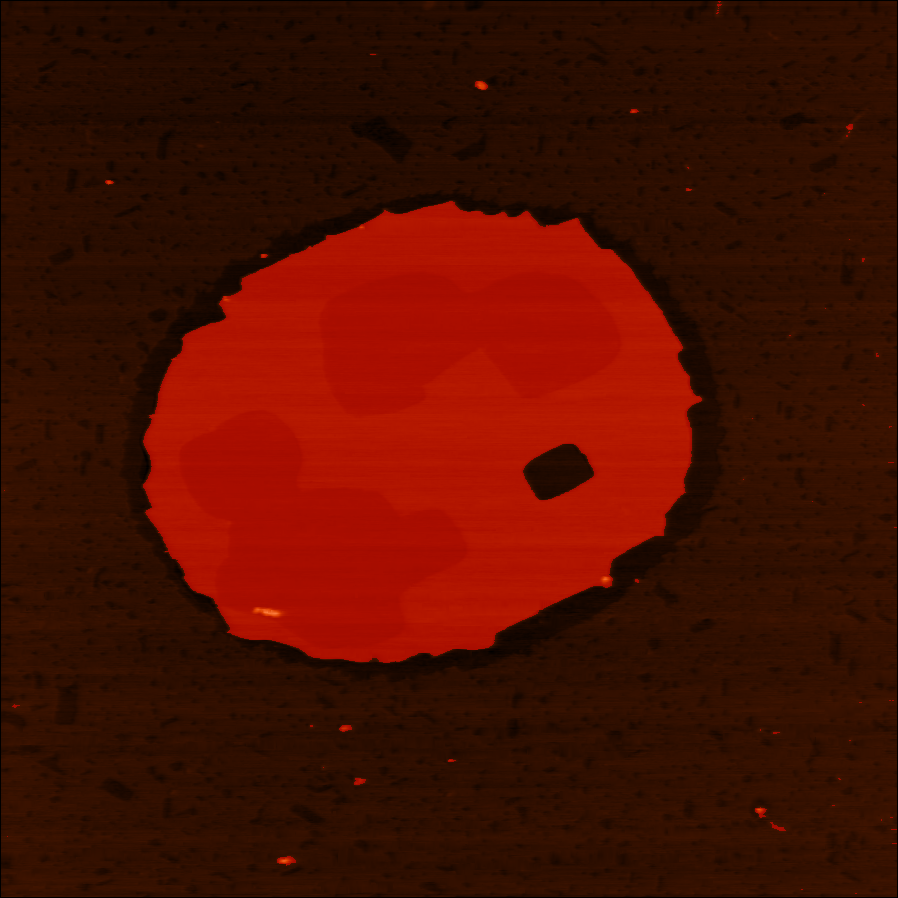

Supplement: Supplementary file 5 — Supplementary Data 3 [file 41467_2019_13488_MOESM5_ESM.tif]
